# Research trends and hotspots in the mental health of widowed older adults: a bibliometric analysis

**DOI:** 10.3389/fpsyt.2025.1612813

**Published:** 2025-07-03

**Authors:** Doudou Lin, Jiaojiao Wu, Weibing Chen, Xiangying Shen, Zhongxiang Cai, Nian Wang, Dou Fu, Yinglin Li, Xiaojie Ma

**Affiliations:** ^1^ Department of Geriatrics, Renmin Hospital of Wuhan University, Wuhan, China; ^2^ Department of Nursing, Renmin Hospital of Wuhan University, Wuhan, China

**Keywords:** widowhood, mental health, bibliometric analysis, older adults, Citespace, VOSviewer

## Abstract

**Background:**

The mental health of widowed older adults has garnered increasing research attention due to its profound impact on well-being and quality of life. Despite growing scholarly interest, a comprehensive bibliometric analysis of evolving research trends, key topics, and knowledge structures remains scarce. This study aims to identify key research themes, emerging trends, and interdisciplinary linkages to inform future studies on the mental health of widowed older adults.

**Methods:**

A bibliometric analysis was conducted using data from the Web of Science Core Collection (2004–2024). CiteSpace, VOSviewer, and the R package “Bibliometrix” were utilized to visualize publication trends, country and author collaborations, keyword co-occurrences, theme analysis, and emerging research topics.

**Results:**

A total of 891 articles were analyzed. The United States produced the highest number of publications, followed by China and the United Kingdom, with the United States, England, and Canada exhibiting strong research collaborations. Depression, prevalence and mental health were identified as core research themes, while life satisfaction and social support emerged as growing areas of interest. Citation burst and thematic evolution analyses revealed shifting scholarly interest from clinical and diagnostic concerns towards psychosocial adaptation and person-centered approaches over time.

**Conclusion:**

This bibliometric study systematically maps the research landscape, hotspots, and emerging trends in the mental health of widowed older adults over the past two decades. The findings provide valuable insights for researchers seeking to identify key research directions, foster interdisciplinary collaborations, and develop targeted interventions to support the mental well-being of widowed older adults.

## 1 Introduction

The global trend of population aging is accelerating. According to the World Health Organization, the number of people aged 60 and above in the world reached 1 billion in 2020 and is projected to double to 2 billion by 2050 (1). As the proportion of older adults increases, the number of widowed older adults is also rising (2). It is estimated that approximately 350 million people were widowed by 2020, of whom approximately 80% were female (3).The significant increase in the population of widowed older adults is evident. In high-income countries, widowhood exhibits high prevalence and gender disparities—data from the U.S. Health and Retirement Study database show that 41.4% of older adults have experienced widowhood (4); among individuals aged 85+ in the UK, 76% of women and 35% of men have been widowed (5). Additionally, a Swedish open-cohort study based on total population registers (including 1,842,487 married individuals aged 60–89 between 2007 and 2016) observed 239,276 transitions to widowhood during the study period (6), further confirming the scale of widowed older adult populations in high-income countries.

Developing countries also face challenges posed by a large base population of widowed individuals. For example, data from China’s Sixth National Population Census indicate that the number of widowed older adults reached 47.74 million, accounting for 26.89% of the total older adult population (7). This statistic not only reflects China’s large elderly population but also highlights the unique needs of widowed groups within social support systems. Concurrently, in rural China, weakened social and emotional support networks due to population migration and evolving family structures have further exacerbated the vulnerabilities of widowed older adults (8). This global shift in marital status reflects profound demographic changes and underscores the urgent need for research to explore its impact on older adults’ well-being, particularly in the domain of mental health.

Spousal loss, as a pivotal life event, exerts long-term and complex effects on the mental health of older adults. The mental health effects of widowhood have garnered significant scholarly attention. Research has linked widowhood to social isolation, negative emotions—including anxiety (9, 10), depression (11–14), and loneliness (15, 16)—as well as impaired sleep quality in older adults (17). A systematic review and meta-analysis estimated the combined prevalence of anxiety disorders at 26.9% (9). A study from China suggests that anxiety symptoms may serve as a key intervention target for widowed older adults (10). Depression is a prevalent psychological condition among widowed older adults. A meta-analysis reported a combined prevalence of 40.6% (33.6%–47.6%) (n=30) using screening scales, whereas studies employing full diagnostic criteria estimated a prevalence of 19.2% (13.4%–25.0%) (n=12) (9). Depression incidence peaked in the first month following spousal loss, with prevalence remaining elevated for at least five years (12, 14).

The psychological impact of spousal loss is long-lasting. Research on grief trajectories among widowed individuals identified four patterns: resilience (54.6%), chronic grief (23.7%), de-pression improvement (11.6%), and chronic depression (10.1%) (11). Over time, women exhibited better recovery patterns, demonstrating greater resilience and improved coping with depression following spousal loss (12, 13). Loneliness poses a significant psychological challenge following spousal loss. Loneliness tends to peak immediately after spousal loss, though research on its long-term effects remains inconclusive (15). Spousal loss significantly alters social networks, heightening the risk of social isolation. This serves as a risk factor for the development of depressive symptoms (15). Additionally, studies indicate that spousal loss negatively affects sleep quality, contributing to emotional distress (17).

Despite existing evidence linking widowhood to anxiety, depression, and loneliness, a systematic mapping of the global research landscape remains lacking. Most studies focus on case interventions or regional surveys, neglecting quantitative analysis of two-decade research trends, core knowledge clusters, and interdisciplinary connections. Addressing this gap is critical for identifying priority research areas and informing the development of targeted mental health interventions.

While traditional systematic reviews are effective for qualitative synthesis, they are constrained by subjective literature selection and narrative bias, which hinder the comprehensive revelation of disciplinary evolution. In contrast, bibliometric analysis using tools such as CiteSpace, VOSviewer, and the R package “Bibliometrix” (18) offers unique methodological advantages: it provides interdisciplinary analytical perspectives by objectively quantifying research output distribution, institutional collaboration networks, and the evolution of research hotspots. The data-driven objectivity and visual analytical capabilities of this approach enable macro-level deconstruction of disciplinary knowledge systems, whose value has been validated across multiple fields (19–22).

Building on these advantages, this study employs a tripartite bibliometric analysis framework to address gaps in research on the mental health of widowed older adults, including the lack of global trend analysis, interdisciplinary knowledge integration, and analysis of regional disparities in research capacity. The study will identify foundational literature and theoretical pathways through co-citation analysis, uncover emerging themes via keyword clustering, and reveal research capacity differences through collaboration network mapping. The innovation of bibliometrics lies in its data-driven objectivity and visual analytic power, enabling macro-level analysis of know. In conclusion, this study mainly answers the following three question:

RQ1: What is the current research status on the mental health of widowed elderly people from 2004 to 2024, from the perspectives of publication trends, journals, countries or regions, and institutions?

RQ2: What are the research hotspots, current research status, and how has the thematic focus evolved in mental health studies on widowed elderly people from 2004 to 2024?

RQ3: What are the development trends and possible future research agendas for the topic of mental health in widowed elderly people, based on the understanding of the current research and development process?

## 2 Methods

### 2.1 Data sources and search strategies

**Figure 1:**
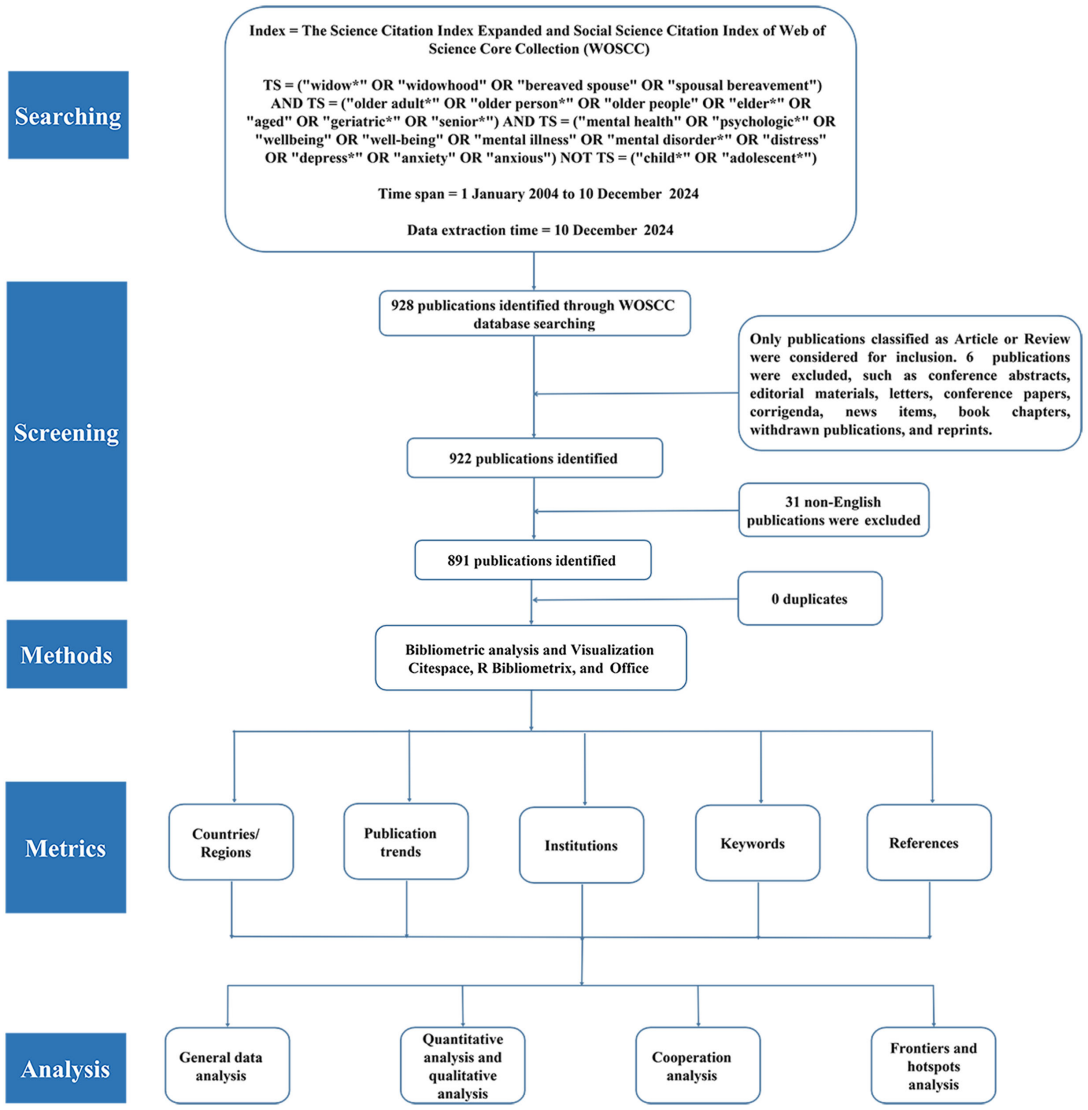
Flowchart of the article selection and analysis process.

This study employed the Web of Science Core Collection (WoSCC) as the primary data source, specifically retrieving records from the Science Citation Index Expanded (SCIE) and the Social Sciences Citation Index (SSCI). These two sub-databases were selected to ensure comprehensive coverage, given that research on the mental health of widowed older adults spans both biomedical and social science domains (23). WoSCC is widely recognized as a reliable database for bibliometric analyses due to its structured and high-quality metadata (24, 25), as well as its compatibility with mainstream bibliometric tools, which minimizes the need for data format conversion and reduces the risk of data loss or corruption. The search strategy was developed based on clinical expertise, relevant literature, Medical Subject Headings (MeSH), and previously published studies (26–31). The final search query was as follows: TS = (“widow*” OR “widowhood” OR “bereaved spouse” OR “spousal bereavement”) AND TS = (“older adult*” OR “older person*” OR “older people” OR “elder*” OR “aged” OR “geriatric*” OR “senior*”) AND TS = (“mental health” OR “psychologic*” OR “wellbeing” OR “well-being” OR “mental illness” OR “mental disorder*” OR “distress” OR “depress*” OR “anxiety” OR “anxious”) NOT TS = (“child*” OR “adolescent*”) The search covered publications from January 1, 2004, to December 10, 2024. The starting year was determined based on methodological considerations rather than a sudden increase in publication volume. A 20-year time frame (2004–2024) was selected to ensure a sufficiently long observation period for capturing longitudinal trends and thematic evolution. This duration balances comprehensiveness and relevance, and it aligns with common practices in bibliometric research (31, 32). Document types were restricted to articles and review articles to ensure the inclusion of substantial scholarly contributions. Publications classified as conference abstracts, editorials, letters, proceedings papers, corrections, news items, book chapters, withdrawn articles, and reprints were excluded. The language was limited to English. The initial search retrieved 928 records. After excluding 31 non-English publications and 6 records that did not meet the document type criteria, 891 eligible articles were retained. To assess the validity of the search strategy, two authors independently reviewed the titles, abstracts, and keywords of the top 100 most-cited publications. Discrepancies were resolved through discussion with a third reviewer. Validation results indicated that 95% of the top-cited records were directly relevant to the research topic, thereby confirming the adequacy of the search terms and strategy (33). All searches and data extraction procedures were completed on December 10, 2024, to prevent potential bias caused by subsequent database updates. After deduplication using CiteSpace, a final dataset of 891 publications was retained for analysis. The study followed established bibliometric methodologies that are widely applied across scientific disciplines. To ensure reproducibility and objectivity, document selection was based solely on predefined inclusion criteria and metadata filtering, without additional manual screening. This approach is consistent with practices reported in recent high-quality bibliometric studies and is considered appropriate for large-scale bibliometric analyses (19, 34–36). The literature selection process is illustrated in **Figure 1**.

### 2.2 Data collection and analysis

Relevant literature was retrieved from the Web of Science Core Collection (WoSCC) and exported in plain text format with full records and cited references. The data were then imported into Microsoft Excel 2021 to establish a bibliometric database. Journal impact factors and H-index values were obtained from the Web of Science platform. The H-index, a widely used metric, captures both the productivity and impact of academic output. An H-index of N signifies that a scholar has published N papers, each cited at least N times, thus reflecting sustained scholarly influence (37). A comprehensive suite of bibliometric analyses was performed using CiteSpace (version 6.3.3), VOSviewer (version 1.6.20), and the Bibliometrix R-package (version 4.4.1). These analyses encompassed publication trends, contributions by countries and institutions, journal co-citation networks, keyword co-occurrence, and thematic evolution.

Microsoft Excel 2021 was utilized to organize data and generate visualizations of annual publication counts and proportions.

CiteSpace, developed by Professor Chaomei Chen (38), is a Java-based bibliometric visualization tool widely used for mapping scientific knowledge. Its core analytical techniques include burst detection, betweenness centrality, and heterogeneous network analysis, which enable the identification of research trends, hotspots, and emerging frontiers. In CiteSpace visualizations, each node represents an element such as a country or institution; the node size corresponds to the frequency of occurrence or citation, while the color reflects the time of first appearance. Nodes outlined in purple denote high betweenness centrality values (>0.1), indicating a pivotal role within the research domain and highlighting potential intellectual turning points. In this study, CiteSpace was employed to generate collaboration networks among countries/regions and institutions, keyword co-occurrence clusters, reference burst detection, and dual-map overlays of journals. The key parameter settings were: time span = 2004–2024; time slicing = 1 year; LRF = 2.5; LBY = 5; e = 1.0; with no pruning applied.

VOSviewer, developed by van Eck and Waltman at the Centre for Science and Technology Studies (CWTS), Leiden University (39), was employed to construct co-citation networks of academic journals and term co-occurrences. This tool excels at producing clear and intuitive visualizations of bibliometric networks. In the resulting maps, nodes are represented as labeled spheres; node size is proportional to citation counts, colors denote clusters, and the thickness of connecting lines reflects the strength of co-occurrence relationships.

Bibliometric analyses were further conducted using Bibliometrix, an open-source R package (version 4.4.1) designed for comprehensive science mapping and bibliometric visualization (18). In this study, Bibliometrix was utilized for country collaboration analysis, thematic evolution, and visualization of Bradford’s Law to assess the concentration of scholarly output. To capture longitudinal trends and thematic shifts, the publication timeline was divided into three distinct sub-periods: 2004–2011, 2012–2016, and 2017–2024.

The integration of CiteSpace, VOSviewer, and Bibliometrix enabled a multidimensional, reproducible exploration of the research landscape in this field. Moreover, to complement the quantitative findings, a critical reading of highly influential studies was conducted to provide nuanced insights and contextual understanding of key contributions (35).

### 2.3 Research ethics

This study does not involve any animal or human trials; therefore, approval from an ethics committee was not required.

## 3 Result

### 3.1 General data

**Figure 2:**
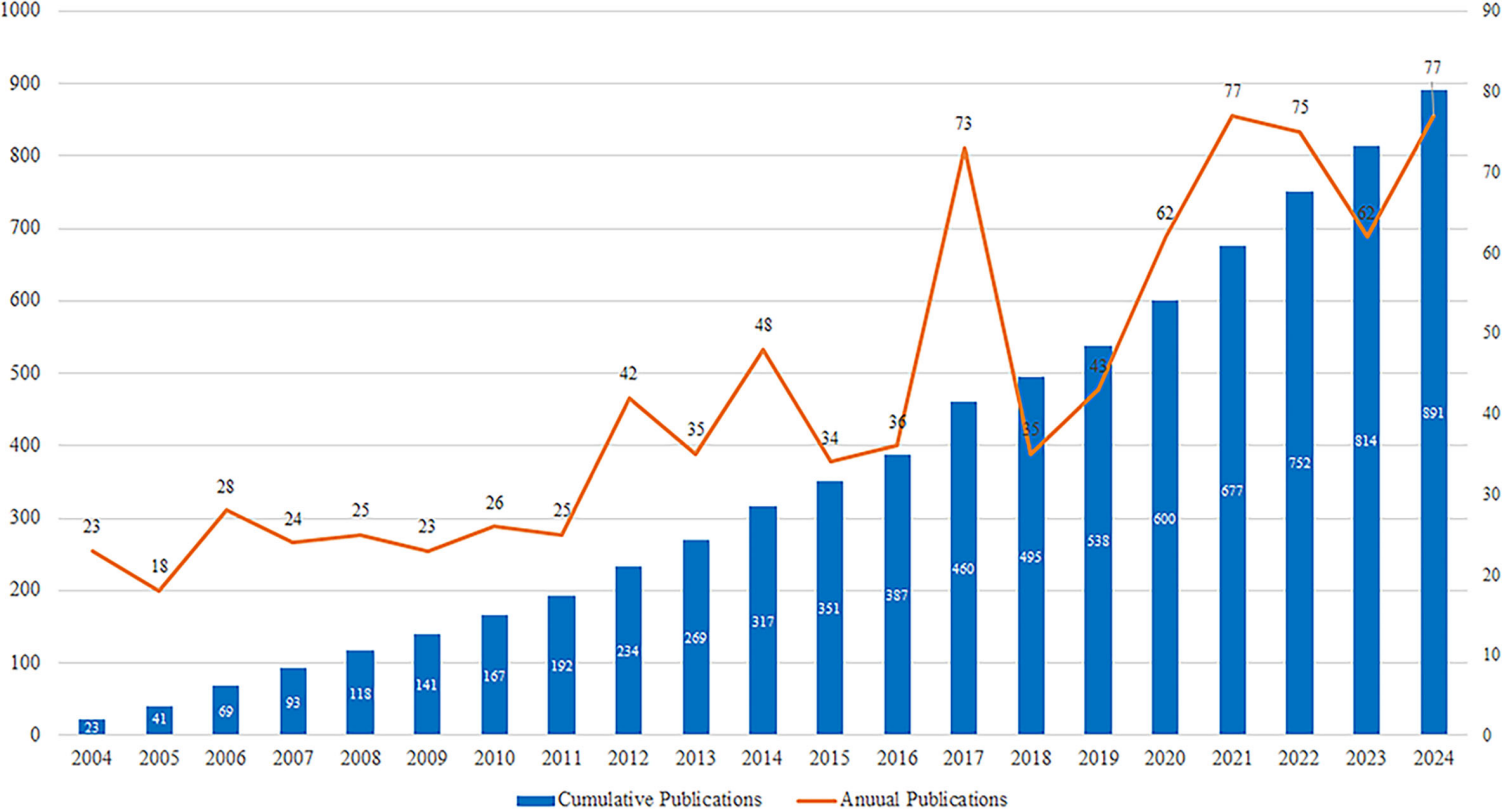
Annual and cumulative output of literature regarding from 2004 to 2024.

A total of 891 articles were retrieved (**Figure 2**). From 2004 to 2024, the annual number of publications exhibited a generally upward trend, despite some year-to-year fluctuations. During the initial phase (2004–2011), annual output remained relatively low, with fewer than 30 publications per year, indicating that research in this field was still in its early exploratory stage. Between 2012 and 2016, the number of publications increased markedly, signaling the beginning of a rapid growth period. In the most recent phase (2017–2024), annual output stabilized at a high level, with more than 75 publications in both 2021 and 2024, each representing approximately 8.64% of the total. This surge in publication volume may be potentially related to the increased attention to the mental health of widowed older adults in the context of the COVID-19 pandemic.

### 3.2 Countries/Regions

**Figure 3:**
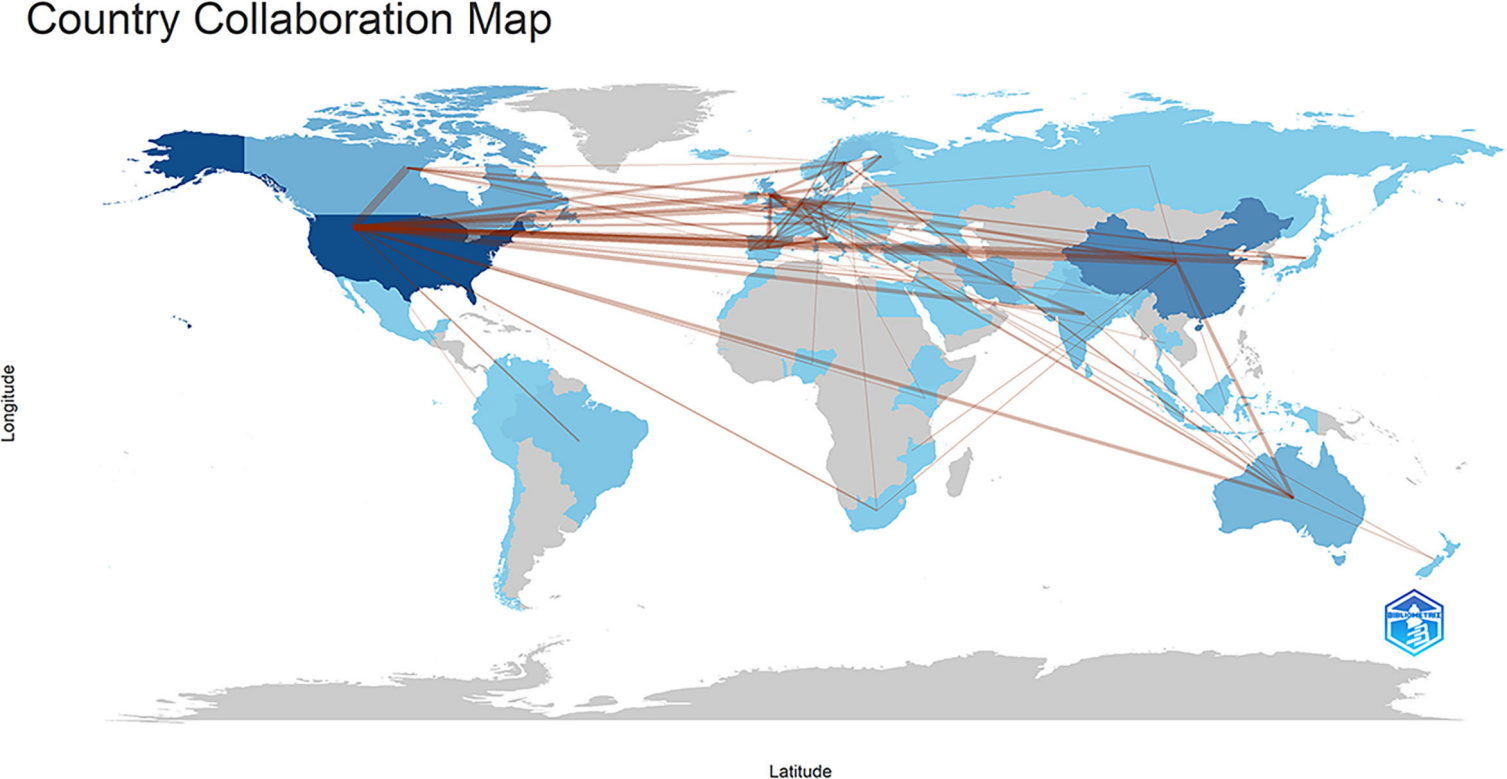
The collaboration map of countries/regions.

**Figure 4:**
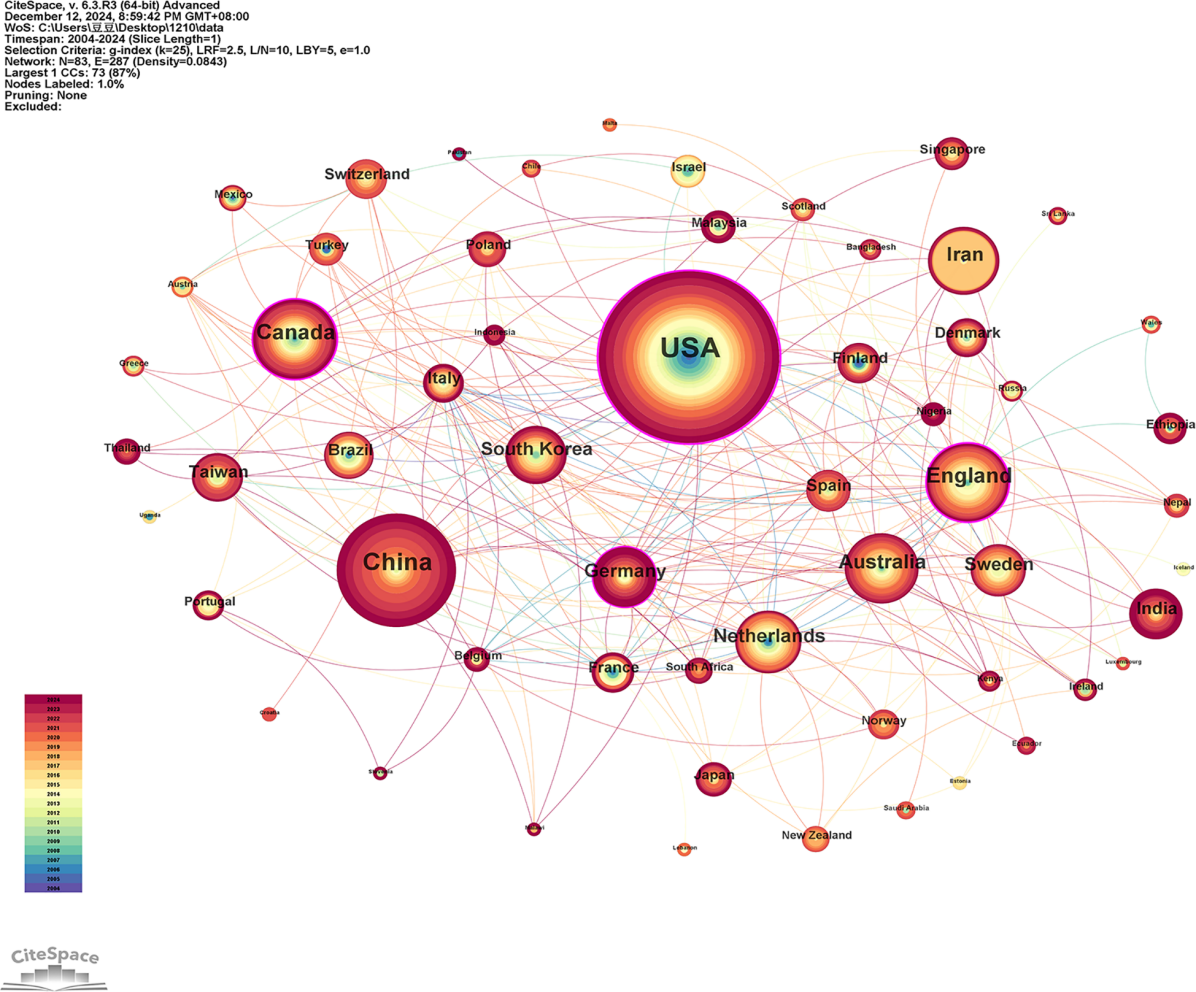
Network visualization of countries/regions, revealing 287 links across 83 countries/regions worldwide.

**Figures 3** and **4** illustrate the distribution and collaboration network of countries/regions in research on the mental health of widowed older adults. Overall, studies are predominantly concentrated in North America, Europe, and Asia, with the United States, the United Kingdom, and China as the most active contributors. A total of 83 countries/regions have published papers in this field. As shown in **Table 1**, the United States ranks first in publication volume (n = 292, 32.77%), followed by China (n = 131, 14.70%) and Canada (n = 67, 7.52%). However, publication quantity alone does not fully capture academic influence. Therefore, betweenness centrality (BC) was also examined to assess each country’s role in international cooperation. The United States (BC = 0.43), the United Kingdom (BC = 0.35), and Canada (BC = 0.23) exhibited relatively high centrality, indicating that these countries not only produce a large volume of publications but also play pivotal roles in global collaboration networks. Additionally, academic impact was evaluated using the H-index and average citations per paper (AC/P). The United States held the highest H-index (55), followed by the United Kingdom (30) and China (28). Regarding AC/P, England (52.71), Germany (45.74), and the United States (42.76) ranked highest, reflecting broader citation influence. In contrast, China (25.32) and South Korea (28.94) demonstrated relatively lower citation averages despite higher publication counts, suggesting potential for improvement in international recognition and research impact. These findings indicate that developed countries dominate not only in productivity but also in collaboration strength and academic influence, while emerging countries such as China and South Korea are increasingly active but may need to further enhance research quality and global visibility.

**Table 1:** Top 10 productive countries/regions ranked by the number of publications.

Ranking	Country/Region	Count	Percentage	BC	H-index	AC/P
1	USA	292	32.77%	0.43	55	42.76
2	China	131	14.70%	0.02	28	25.32
3	Canada	67	7.52%	0.23	22	21.12
4	England	65	7.30%	0.35	30	52.71
5	Australia	47	5.27%	0.09	18	18.11
6	Iran	44	4.94%	0.02	7	14.73
7	Netherlands	42	4.71%	0.09	26	42.69
8	Germany	39	4.38%	0.11	16	45.74
9	South Korea	35	3.93%	0.00	17	28.94
10	Sweden	32	3.59%	0.09	14	41.25

### 3.3 Institutions

**Figure 5:**
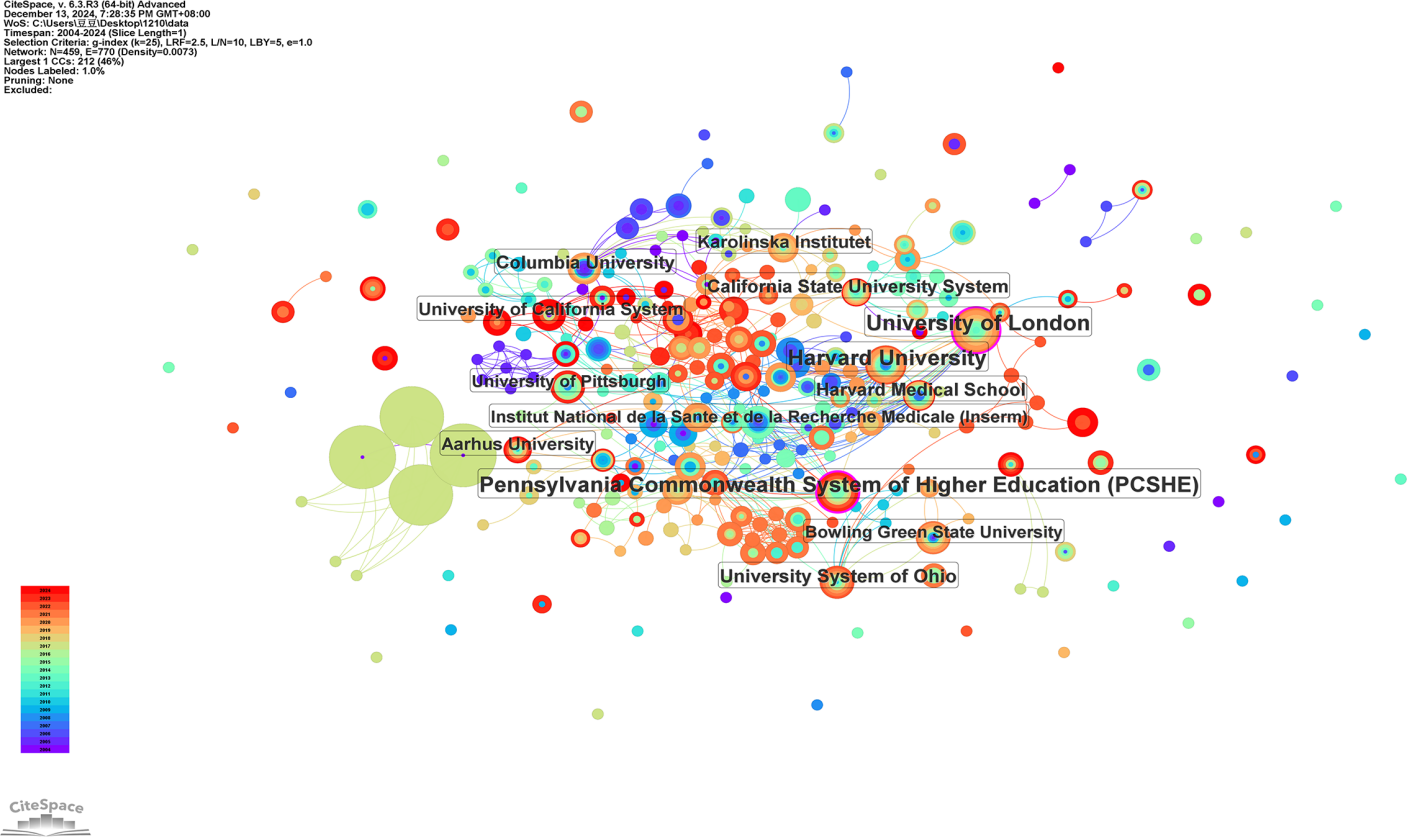
Network visualization of institutions, produced using CiteSpace, showing 770 links across 459 institutions globally.

A total of 1,521 institutions have contributed to research on the mental health of widowed older adults. Based on CiteSpace analysis, 729 institutions were identified and visualized within the collaboration network (**Figure 5**). **Table 2** lists the top 10 institutions by publication volume, including five from the United States, four from Iran, and one from the United Kingdom. The Ministry of Health and Medical Education (MOHME) and Tehran University of Medical Sciences lead with 34 publications each (3.82%), followed by Shahid Beheshti University of Medical Sciences and Iran University of Medical Sciences, both with 32 publications (3.59%). Network visualization (**Figure 5**) reveals low centrality across global institutional collaborations, with only two institutions showing notable betweenness centrality: the University of London (BC = 0.10) and the Pennsylvania Commonwealth System of Higher Education (PCSHE) (BC = 0.12). This suggests that institutional collaboration remains relatively fragmented, highlighting the need to strengthen inter-institutional cooperation to further advance the field. Regarding academic impact, the H-index ranking identifies the University of London (17), Harvard University (15), and the University System of Ohio (14) as leading institutions. Moreover, average citations per publication (AC/P) indicate that Harvard Medical School (65.85) and Harvard University (58.25) achieve the highest citation impact per article, underscoring the significant influence of their research outputs and the value of their research and collaboration models.

**Table 2:** Top 10 institutions ranked by publication productivity.

Ranking	Institution	Count	Percentage	BC	H-index	AC/P
1	Ministry of Health & Medical Education (MOHME)	34	3.82%	0.00	4	11.18
2	Tehran University of Medical Sciences	34	3.82%	0.00	5	15.53
3	Shahid Beheshti University Medical Sciences	32	3.59%	0.00	3	9.61
4	Iran University of Medical Sciences	32	3.59%	0.00	3	6.97
5	University of London	22	2.47%	0.10	17	46.79
6	Pennsylvania Commonwealth System of Higher Education (PCSHE)	21	2.36%	0.12	12	33.04
7	Harvard University	21	2.36%	0.09	15	58.25
8	University System of Ohio	13	1.46%	0.05	14	31.48
9	California State University System	11	1.23%	0.04	6	26.45
10	Harvard Medical School	11	1.23%	0.00	11	65.85

### 3.4 Journals

Journals play a crucial role as platforms for disseminating high-quality academic research, with their influence often measured by publication volume and citation counts within a specific field.

**Figure 6:**
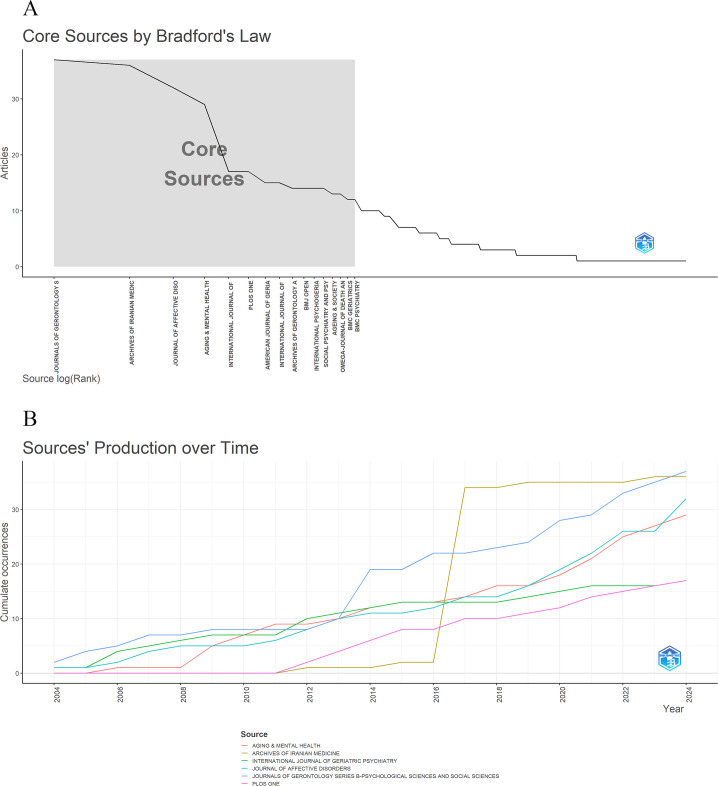
**(A)** Core journals identified based on Bradford’s Law. **(B)** Cumulative publication growth of the top six contributing journals from 2004 to 2024.

**Figure 7:**
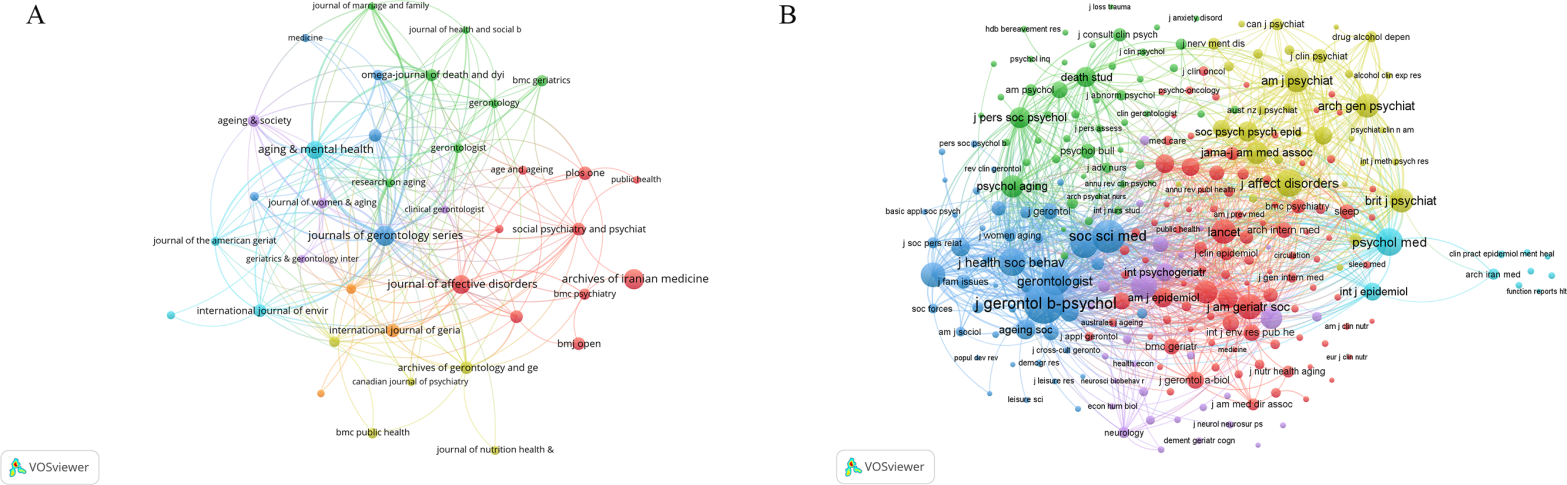
**(A)** Network map of scholarly journals. **(B)** Network map of co-cited scholarly journals. (This embedded figure is for review purposes only. A high-resolution version (300 dpi TIFF) has been submitted separately for publication).

This study analyzed 891 articles published across 339 journals. Applying Bradford’s Law—which posits that journals ranked by publication output can be divided into three zones, each contributing roughly one-third of the total articles while the number of journals grows exponentially (1: a: a²) (40) —the journal set was categorized accordingly using the Bibliometrix R package. Zone 1 comprised 16 core journals, Zone 2 included 68 journals, and Zone 3 contained 255 journals, each zone contributing approximately one-third of the total publications (**Figure 6A**, **Table 3**), confirming a concentration of scholarly output within a relatively small subset of journals. From 2004 to 2024, cumulative publication output among the six most prolific journals exhibited a steady upward trend, with a marked acceleration after 2016 (**Figure 6B**). Additionally, a citation network of journals was visualized using VOSviewer (**Figure 7A**). **Table 4** lists the top 10 journals by publication volume, along with key metrics including total citations, average citations per article, journal quartile, and impact factor. Among these, *Journals of Gerontology Series B: Psychological Sciences and Social Sciences* ranked highest in publication volume (n = 37), total citations (1584), and average citations per article (42.81). This journal is classified as Q1 with an impact factor of 4.8. It is followed by *Archives of Iranian Medicine* (n = 36) and *Journal of Affective Disorders* (n = 32). Notably, some journals with relatively lower publication volumes demonstrate high average citation rates, such as *Archives of Gerontology and Geriatrics* (67.29), *Social Psychiatry and Psychiatric Epidemiology* (54.50), and *International Journal of Geriatric Psychiatry* (48.65), most of which are ranked within Q1 or Q2 quartiles, reflecting strong academic quality. Furthermore, journals with moderate or lower publication volumes, such as *PLOS One* and *International Journal of Environmental Research and Public Health*, serve as important multidisciplinary platforms facilitating research exchange in this field.

**Table 3:** According to Bradford’s law, the 339 journals were classified into zones 1–3.

Zone	No. of journals	No. of publications	Percentage
1	16	304	34.1%
2	68	293	32.9%
3	255	294	33.0%
Total	339	891	100.0%

**Table 4:** Top 10 journals ranked by publication productivity.

Ranking	Journal	Document	Total citation	Average citation per paper	Quartile in category	IF
1	Journals of Gerontology Series B-Psychological Sciences And Social	37	1584	42.81	Q1	4.8
2	Archives of Iranian Medicine	36	274	7.61	Q3	1.0
3	Journal of Affective Disorders	32	1373	42.91	Q1	4.9
4	Aging & Mental Health	29	1006	34.69	Q3	2.8
5	International Journal of Geriatric Psychiatry	17	827	48.65	Q2	3.6
6	Plos One	17	615	36.18	Q1	2.9
7	American Journal of Geriatric Psychiatry	15	531	35.40	Q1	4.4
8	International Journal of Environmental Research and Public Health	15	181	12.07	Q2	4.6
9	Archives of Gerontology and Geriatrics	14	942	67.29	Q2	3.5
10	Social Psychiatry and Psychiatric Epidemiology	14	763	54.50	Q1	3.6

The co-citation frequency of a journal reflects its academic impact within a specific research domain (41). Using VOSviewer, we generated a co-citation network map of 266 journals meeting a minimum citation threshold of 20 (**Figure 7B**). This threshold balances the inclusion of influential sources with the need for visual clarity and interpretability. Applying this cutoff excludes journals with low co-citation counts that may introduce noise or marginal relevance, thereby emphasizing core journals with substantial scholarly impact. **Table 5** lists the top 10 most frequently co-cited journals, led by The Journals of Gerontology: Series B, Social Science & Medicine, and The Gerontologist. Notably, eight of these journals fall within the Q1 quartile, underscoring their prominent academic influence in mental health research among widowed older adults.

**Table 5:** Top 10 most co-cited journals ranked by citation count.

Ranking	Journal	Citations	Total link strength	Quartile in category	IF
1	The Journals of Gerontology: Series B, Psychological Sciences and Social Sciences	1080	37904	Q1	4.8
2	Social Science & Medicine	697	23452	Q1	4.9
3	Gerontologist	546	18281	Q1	4.6
4	Journal of Affective Disorders	502	16395	Q1	4.9
5	Journal of Health and Social Behavior	496	17590	Q1	6.3
6	Aging & Mental Health	491	17031	Q3	2.8
7	Psychological Medicine	485	15301	Q1	5.9
8	International Journal of Geriatric Psychiatry	455	15524	Q2	3.6
9	Journal of the American Geriatrics Society	414	12317	Q1	4.3
10	Lancet	406	11267	Q1	98.4

**Figure 8:**
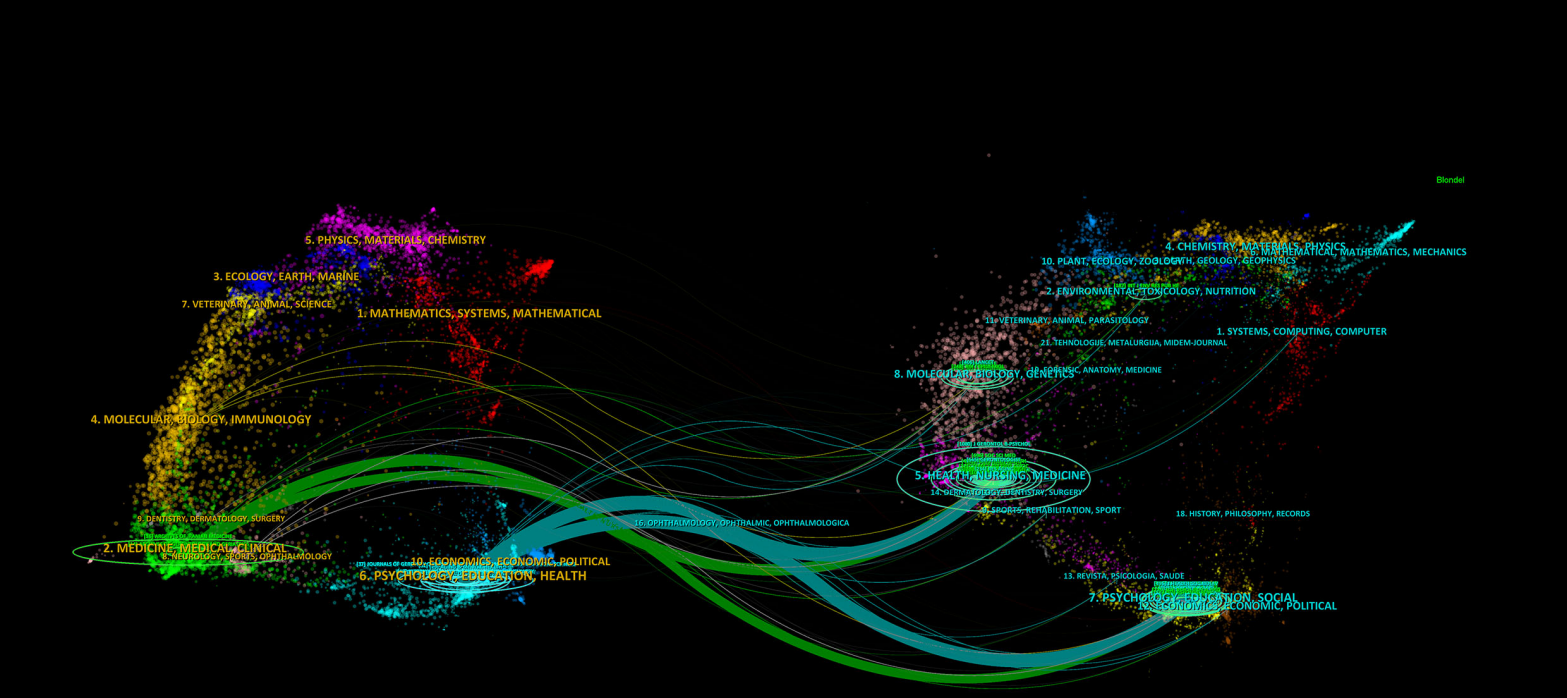
Dual-map overlay analysis mapping.

CiteSpace was employed to conduct a dual-map overlay analysis of journal fields (**Figure 8**), illustrating the interconnections between citing and cited journals across research domains. The left side represents citing journal clusters, while the right side shows cited journal clusters, with colored lines denoting citation relationships. The map was clustered using the Z-Score algorithm, revealing two primary citation paths. Within the cited clusters, “Health, Nursing, Medicine” and “Psychology, Education, Sociology” were most prominent. The main citation paths originate from these clusters: the green path indicates that journals categorized as “Medicine, Medical, Clinical” predominantly cite these clusters, whereas the turquoise path shows that journals classified under “Psychology, Education, Health” similarly reference them. These citation trajectories highlight the interdisciplinary nature of research on the mental health of widowed older adults, bridging both health sciences and social sciences.

### 3.5 Co-cited references and burst references

**Table 6** lists the top five most co-cited references, which primarily focus on key topics such as widowhood and depression, cognitive status assessment, and post-bereavement health outcomes. The most co-cited reference is Radloff, L. S (42) “The CES-D Scale: A Self-Report Depression Scale for Research in the General Population,” cited 96 times. This study introduced the CES-D scale, a widely adopted instrument for assessing depressive symptoms in older adults. The second most co-cited reference is Umberson D.’s “Widowhood and Depression: Explaining Long-Term Gender Differences in Vulnerability” (43), cited 62 times. This research explores the long-term impact of widowhood on depression, highlighting gender differences as a key factor, and provides a foundational basis for understanding gender-specific vulnerability to depressive symptoms following widowhood.

**Table 6:** Top 5 co-cited references.

Ranking	Year	First Author	Reference	Journal	Co-citations
1	1977	Radloff, L. S. (42)	The CES-D Scale: A Self-Report Depression Scale for Research in the General Population	Applied Psychological Measurement	96
2	1992	Umberson, D. (43)	Widowhood and Depression: Explaining Long-Term Gender Differences in Vulnerability	Journal of Health and Social Behavior	62
3	1975	Folstein, M. F.	Mini-Mental State: A Practical Method for Grading the Cognitive State of Patients for the Clinician	Journal of Psychiatric Research	60
4	2014	Sasson, I. (3)	Widowhood and depression: new light on gender differences, selection, and psychological adjustment	The Journals of Gerontology: Series B, Psychological Sciences and Social Sciences	50
5	2007	Stroebe, M.	Health Outcomes of Bereavement	The Lancet	50

**Figure 9:**
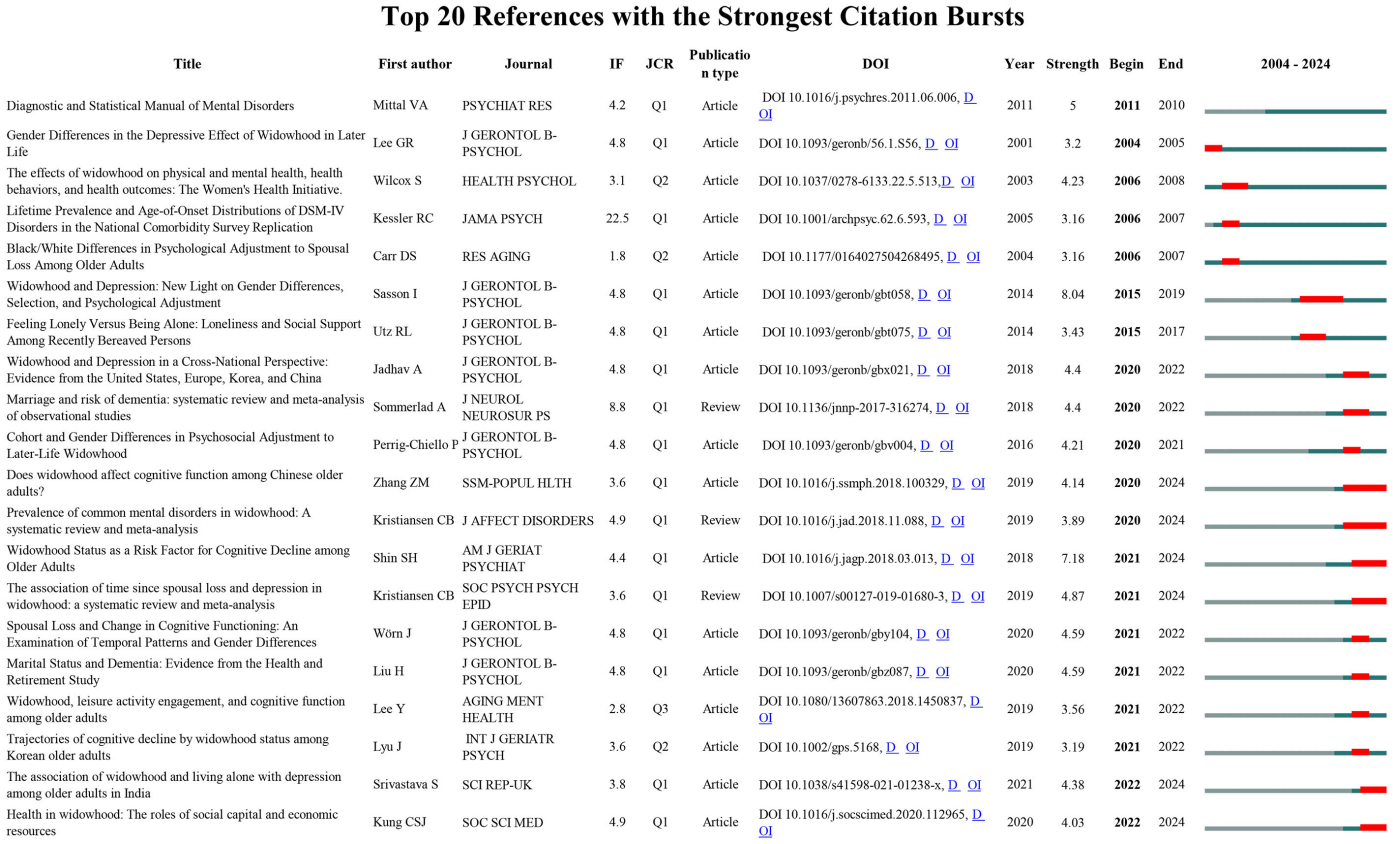
Top 20 references with the strongest citation bursts.

Using CiteSpace, we identified 20 references exhibiting significant citation bursts, indicating their substantial influence on subsequent research and reflecting widespread academic attention in the field. **Figure 9** depicts the burst periods for each reference, with red bars highlighting the years of peak citation activity. The top three references by burst intensity are: Sasson & Umberson (2014) (3), Widowhood and Depression: New Light on Gender Differences, Selection, and Psychological Adjustment (burst intensity = 8.04), which explores the impact of widowhood on depression, particularly focusing on the role of gender differences; Shin et al. (2018) (44), Widowhood Status as a Risk Factor for Cognitive Decline among Older Adults (burst intensity = 7.18), which examines the association between widowhood and cognitive decline in older adults; Mittal & Walker (2011) (45), Diagnostic and Statistical Manual of Mental Disorders (burst intensity = 5), a key diagnostic manual in the mental health field, which highlights the strong link between mental health diagnoses and widowhood-related psychological health research.

The current burst analysis reveals several prominent research hotspots in the mental health of widowed older adults, focusing on gender differences, depression, cognitive function, and social factors. Two meta-analyses by Blanner Kristiansen et al. (2019) and Kristiansen et al. (2019) (9, 46) have provided comprehensive evidence on the incidence of mental disorders after widowhood and the relationship between widowhood duration and depression severity. In addition, Shin et al. (2018) (44) investigated the association between widowhood and cognitive decline, demonstrating a significant impact with a burst intensity of 7.18. Furthermore, Kung (2020) (47) highlighted the critical role of social capital and economic resources in promoting psychological well-being among widowed individuals, underscoring the importance of social support and financial stability in mitigating mental health risks.

### 3.6 Keyword visualization and burst test

**Figure 10:**
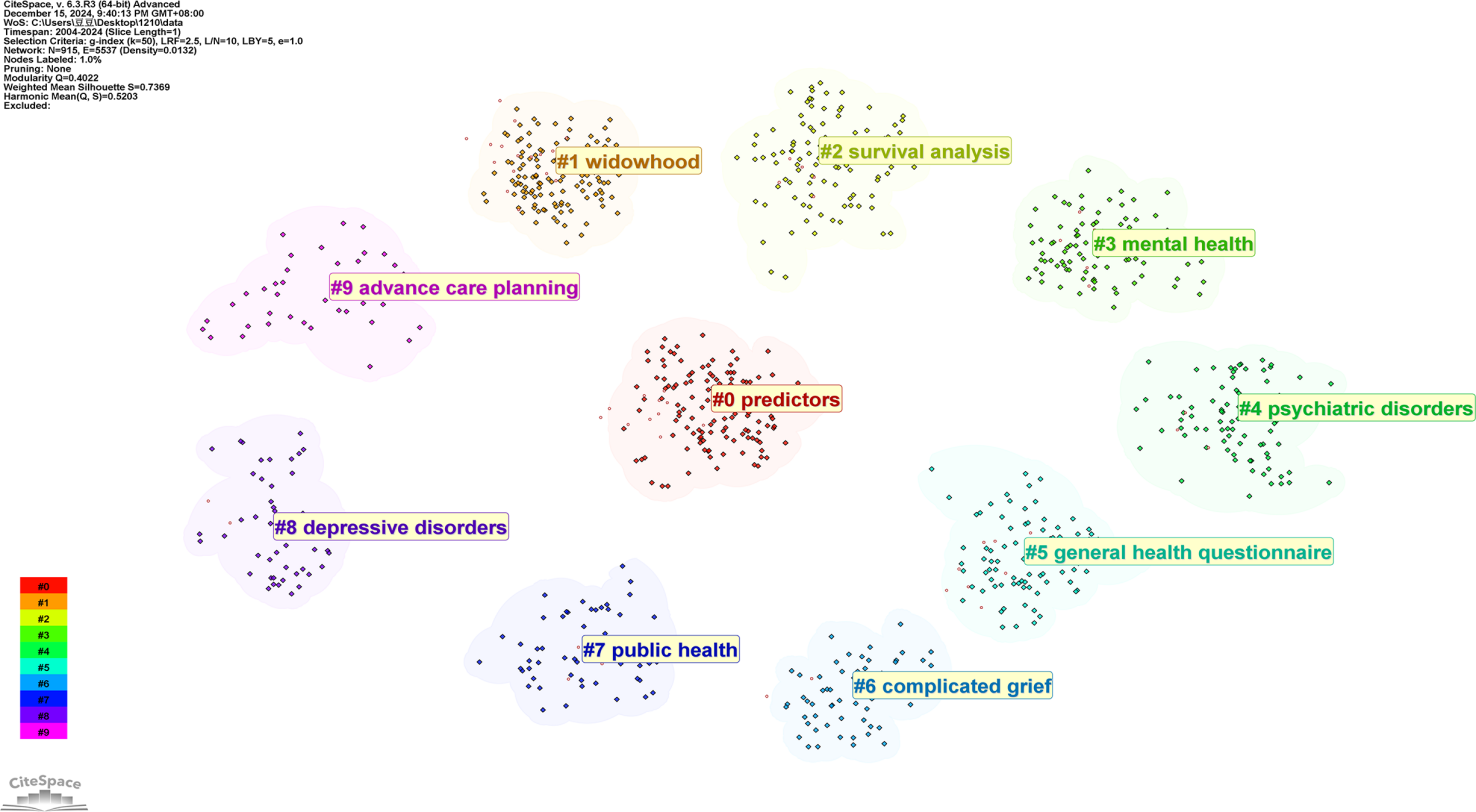
Cluster analysis of keyword-related research networks.

Keywords represent the core concepts of research articles, and their analysis facilitates the identification of focal points within a given field. **Table 7** lists the top ten most frequently occurring keywords alongside their centrality scores. Among these, “health,” “older adults,” and “depression” rank highest in frequency, while “mental health,” “older adults,” and “prevalence” exhibit the greatest centrality, indicating their pivotal roles in the intellectual structure of the field. To further elucidate this structure, keyword clustering analysis was conducted using CiteSpace. During network construction and node selection, a range of K-values (as the scaling factor in the g-index) was tested to optimize the balance between thematic granularity and interpretability. Lower K-values yielded numerous narrowly defined clusters that impeded thematic synthesis. Specifically, clustering results were compared across multiple K-values (e.g., 20, 30, 40, 50, 60) and evaluated using both quantitative metrics—modularity Q-value and mean silhouette S-value—and qualitative assessment of thematic coherence. Among these, K = 50 provided the optimal trade-off, achieving satisfactory structural modularity and semantic clarity. Although previous studies have employed similar parameter ranges, our selection was validated internally to ensure robustness. In the resulting clustering map, clusters with lower numerical labels (e.g., #0, #1) generally correspond to larger or more influential thematic domains (34). Clustering quality was assessed using two widely accepted structural metrics: a modularity Q-value above 0.3 indicates a significant community structure, while a silhouette score exceeding 0.5 (ideally > 0.7) reflects high intra-cluster homogeneity and clear inter-cluster separation (38). In our analysis, the clustering yielded a modularity Q-value of 0.402 and a silhouette score of 0.737, indicating that the identified thematic groups are both structurally well-defined and internally consistent. The integration of these quantitative metrics with expert qualitative evaluation supported the selection of K = 50 as the optimal parameter. This approach allowed us to capture the most salient and recurrent research themes while effectively balancing between over-fragmentation and excessive generalization. As illustrated in **Figure 10**, the clustering process delineated ten distinct thematic groups: #0 predictors, #1 widowhood, #2 survival analysis, #3 mental health, #4 psychiatric disorders, #5 general health questionnaire, #6 complicated grief, #7 public health, #8 depressive disorders, and #9 advance care planning. These clusters comprehensively represent the core research domains encompassing health predictors, mental health challenges (notably complicated grief), and public health interventions pertinent to widowed older adults.

**Table 7:** Top 10 keywords ranked by the number of publications.

Ranking	Keywords	Count	Centrality
1	health	233	0.06
2	older adults	223	0.07
3	depression	186	0.06
4	prevalence	156	0.07
5	mental health	126	0.09
6	widowhood	121	0.03
7	mortality	115	0.03
8	symptoms	101	0.04
9	risk	97	0.05
10	marital status	94	0.04

**Figure 11:**
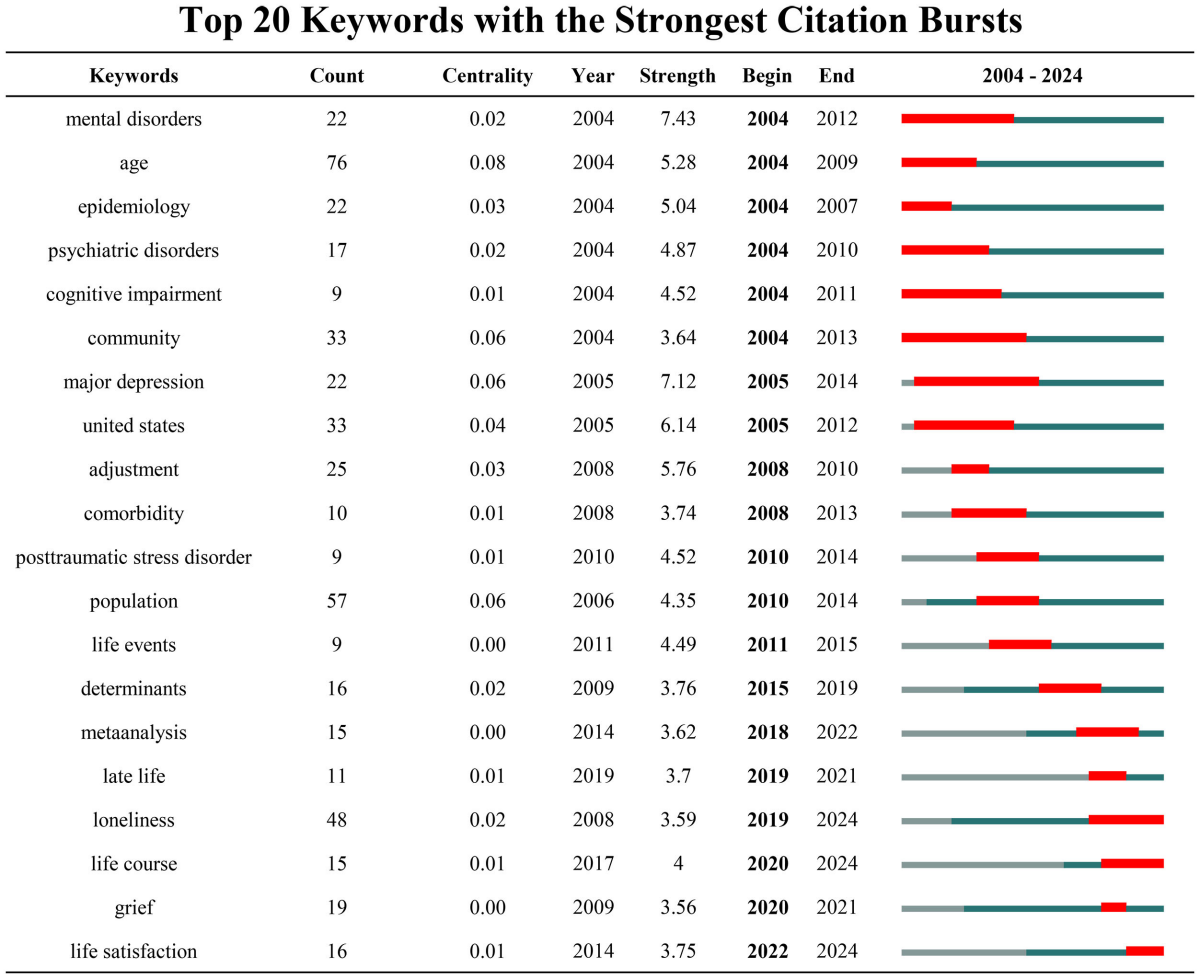
Top 20 keywords with the strongest citation bursts.

Keyword burst refers to keywords that are frequently cited within a specific period. Analyzing keyword bursts helps to identify emerging trends and frontiers in the field. **Figure 11** presents the top 20 keywords with the highest burst strength from 2004 to 2024, with the red sections on the blue timeline indicating the start year, end year, and duration of the burst. It is evident that “mental disorder” (burst strength = 7.43) and “major depression” (burst strength = 7.12) exhibit the highest burst intensity, indicating that mental health issues, particularly depression, remain central to the study of psychological well-being in widowed older adults, especially in the earlier stages of widowhood. Based on the temporal distribution of keyword bursts, research in this field has evolved through three distinct phases: (1) 2004–2011: The focus of research during this period was primarily on psychological disorders and epidemiology, with attention given to mental illnesses and cognitive impairments in the widowed elderly population. (2) 2012–2016: The research gradually shifted towards adaptive processes, exploring how widowed older adults cope with the emotional upheaval associated with the loss of a spouse. Keywords such as “life events,” “post-traumatic stress dis-order (PTSD),” and “psychological adaptation” became prominent during this phase. (3) 2017–2024: Research interests began to expand into emotional and social dimensions, with a growing focus on social support and emotional needs. Keywords such as “loneliness,” “life course,” and “life satisfaction” reflect an increasing emphasis on the social and emotional aspects of widowhood.

### 3.7 Trend topics and thematic evolution

#### 3.7.1 Trend topics

**Figure 12:**
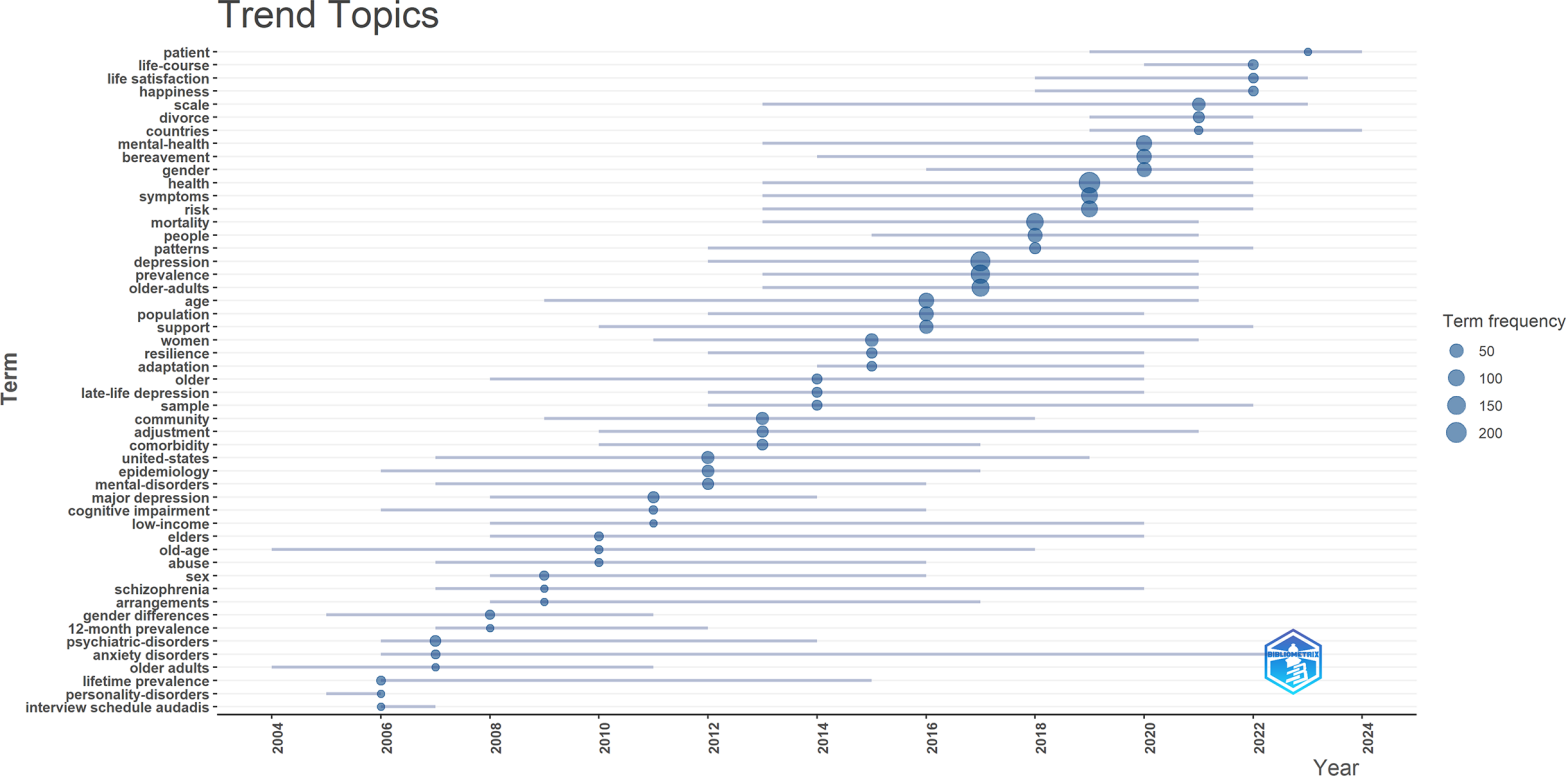
Analysis of trend topics.

Trend topic analysis was conducted to examine emerging research themes and their temporal development. As shown in **Figure 12**, several high-frequency keywords began to surface after 2017. These include terms such as “prevalence,” “patterns,” “mortality,” “risk,” “symptoms,” and “gender,” which indicate the evolving focus areas within this field. In more recent years, additional keywords—such as “countries,” “scale,” “happiness,” “life satisfaction,” “life course,” and “patient”—have gained prominence. Notably, topics such as “life satisfaction” and “life course” align closely with the results of the keyword burst analysis, suggesting a meaningful shift in research orientation. This evolution reflects a transition from a predominantly pathological perspective to a more holistic and human-centered approach, emphasizing quality of life and the dynamic nature of the life course in widowed older adults.

#### 3.7.2 Thematic map

**Figure 13:**
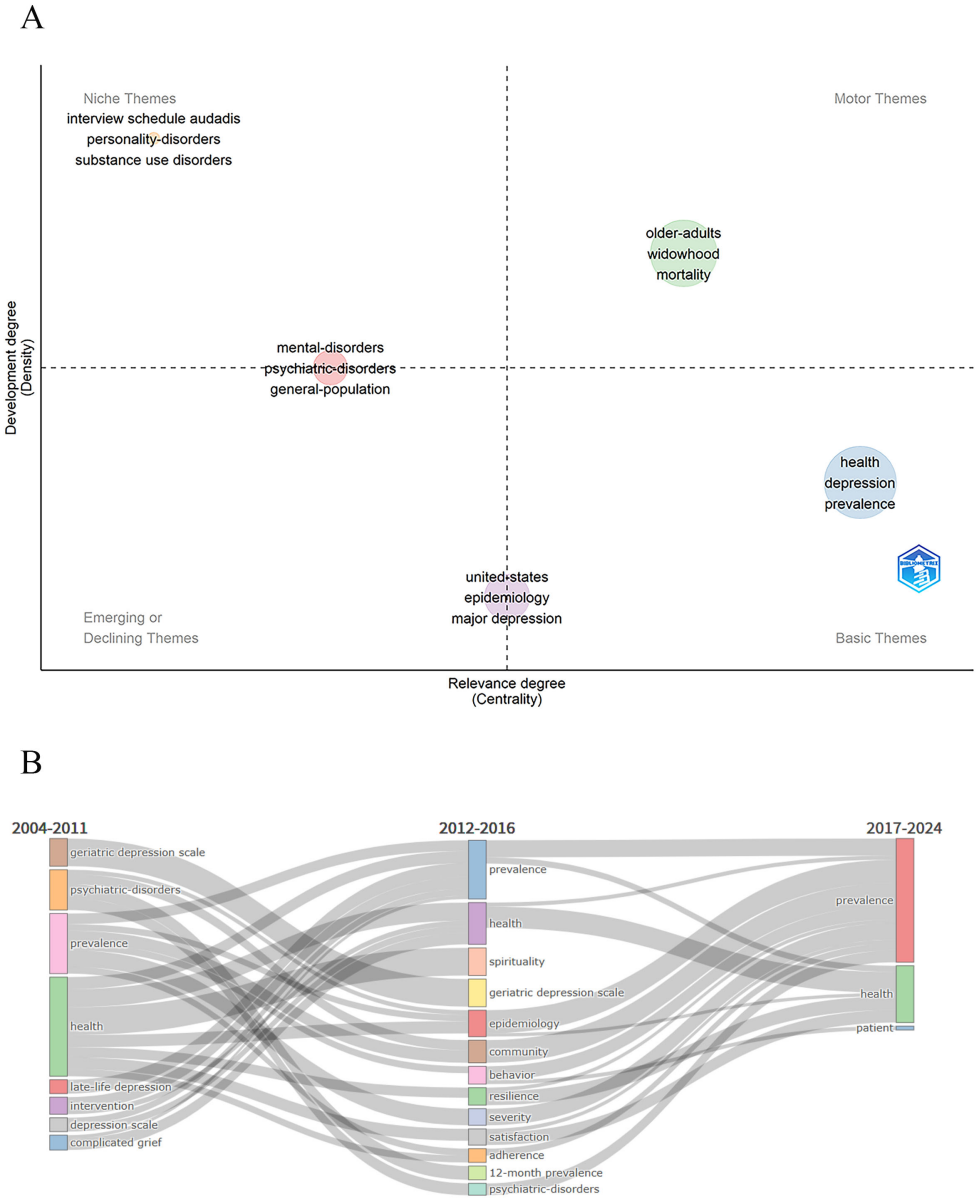
**(A)** Thematic map of 2004–2024. **(B)** Thematic evolution over time.

A thematic map was generated using the Bibliometrix R package to illustrate the conceptual structure of research on the mental health of widowed older adults (**Figure 13A**). Themes were categorized into four quadrants based on their centrality (relevance to the field) and density (level of internal development). Motor themes, such as “older adults,” “widowhood,” and “mortality,” appeared in the upper-right quadrant, reflecting well-developed and central areas of sustained scholarly interest. Basic themes like “health,” “depression,” and “prevalence” were highly relevant yet underdeveloped, suggesting foundational topics with potential for deeper exploration. Niche themes (e.g., “personality disorders,” “substance use disorders”) showed high internal development but limited field relevance. Meanwhile, clusters like “United States,” “epidemiology,” and “major depression” fell into the lower-left quadrant, identifying them as either emerging or declining themes with marginal or shifting research focus. These findings delineate the thematic landscape of the field, highlighting both well-established topics and underexplored areas that merit additional investigation.

#### 3.7.3 Thematic evolution

Thematic evolution analysis (**Figure 13B**) traced the development of research themes on the mental health of widowed older adults across three periods (2004–2024). In the first phase (2004–2011), the focus centered on diagnostic tools (e.g., Geriatric Depression Scale, depression scale) and clinical categorizations (e.g., psychiatric disorders, mental disorders), reflecting a primarily biomedical perspective. During the second phase (2012–2016), the scope broadened to include psychosocial aspects, with emerging themes such as resilience, satisfaction, community, and behavior, alongside sustained interest in health and prevalence, signaling increased emphasis on psychological adaptation and social context. In the most recent period (2017–2024), while foundational themes like health and prevalence persisted, a more person-centered approach became evident, highlighted by the prominence of “patient” as a thematic node. The varying thickness of connecting lines represents the strength of thematic continuity, with thicker links indicating greater conceptual persistence. Overall, the thematic trajectory reveals a shift from diagnostic and epidemiological priorities toward more holistic, human-centered perspectives on mental health in widowed older adults.

## 4 Discussion

### 4.1 General information

Bibliometric was used to analysis to profile the research landscape on the mental health of widowed older adults, revealing that publication from 2004–2024 showed a fluctuating upward trend with a post-COVID-19 surge (average ~75 papers/year), likely linked to pandemic-induced spousal loss among older adults (COVID-19 mortality rate ~5.5%, predominantly in older populations). The U.S. led in publications (twice China’s output and four times Canada’s), with strong collaboration among the U.S., UK, and Canada, while most countries lacked robust international cooperation. Among institutions, U.S. universities dominated the top 10 (5 of 10), though Iranian institutions showed organizational strength for future growth, despite lower overall output; global institutional collaboration remains insufficient. The Journals of Gerontology: Series B-Psychological Sciences and Social Sciences was the most influential journal, with 80% of top 10 journals in JCR Q1, and citation analysis highlighted cross-disciplinary links to psychology, education, and health sciences. These findings outline the overall research distribution and development, addressing RQ1 on publication trends, major contributors, and collaborative patterns. Future research would benefit from enhanced international and institutional collaboration, leveraging funding opportunities and emerging research infrastructure to accelerate progress.

### 4.2 Knowledge base

Papers that are frequently co-cited by scholars constitute the knowledge base of a particular research field. These co-cited studies serve as foundational works, offering a solid basis for advancing new research. Consequently, we analyzed the co-cited literature to assess the knowledge base on the mental health of widowed older adults.

In our study, four of the five most-cited papers discussed depression, suggesting that it is a central theme in the mental health of widowed older adults. Depression is among the most significant psychological challenges faced by older adults following spousal loss, warranting increased awareness and attention. Two main bodies of literature focus on the design and application of assessment tools: one concerns self-report scales for measuring depression, while the other pertains to cognitive ability assessments. Widowed older adults are at a higher risk of cognitive de-cline, driving the development of cognitive assessment tools and advancing research into cognitive function screening. One of the five most-cited literature reviews examines the relationship between bereavement and both physical and mental health outcomes. The review highlights that bereavement is associated with higher mortality rates, reduced physical well-being, and psychological distress during the grieving process.

Throughout the literature, the most-cited articles primarily focus on the development of self-report depression assessment tools for widowed older adults, the creation of cognitive function assessment tools, and the interrelationship between bereavement distress and physical and mental health. These studies not only draw researchers’ attention to the mental health status of widowed older adults but also provide a theoretical foundation for subsequent psychological screening and intervention. These high-impact studies collectively establish a theoretical and methodological framework addressing RQ2 by elucidating core topics and measurement tools critical for assessing widowed older adults’ mental health.

### 4.3 Thematic analysis

This study integrates thematic trend analysis and quadrant diagram analysis to reveal the developmental characteristics of research on the mental health of widowed older adults from temporal and distributional dimensions. The results of these two analyses complement each other, providing a panoramic view of research in this field.

In the upper - right quadrant, highly relevant and rapidly developing themes such as “older - adults,” “widowhood,” and “mortality” echo the findings of the thematic trend analysis. During the initial stage from 2004 to 2011, academic research primarily focused on the health and specific mental issues of widowed older adults, laying the foundation for core thematic research. From 2012 to 2016, the expansion of research on epidemiology and psychological resilience further enriched the connotations of these core themes. Between 2017 and 2024, the increased attention to individual patients reflected the practical application and deepening of core themes. These themes have persisted throughout the research period, continuously guiding the development of the field and forming a stable theoretical and practical system. In the upper - left quadrant, themes like “interview schedule audadis” and “personality - disorders,” despite showing relatively high development levels, have weak associations with the core research area. During the research expansion phase (2012 - 2016), some emerging directions, such as the application of specific assessment tools and studies on psychological disorders in non - widowed contexts, failed to effectively integrate into core topics, leading to research fragmentation. Although these studies have developed in - depth branches locally, their lack of linkage with core themes limits their contribution to the overall development of the field. In the future, cross - thematic integration should be strengthened. The lower - left quadrant features themes with low relevance and development, such as “united - states,” “epidemiology,” and “major depression,” which align with the changing research hotspots identified in the trend analysis. “Epidemiology” emerged as a new focus from 2012 to 2016 but remains underdeveloped. Research on “major depression” may have declined from 2017 to 2024 due to outdated research paradigms. This indicates that researchers should dynamically adjust resource allocation by considering both temporal trends and distribution characteristics, promoting the development of emerging areas and innovating traditional research paradigms. In the lower - right quadrant, themes with high relevance but low development, including “health,” “depression,” and “prevalence,” have persisted throughout the research but their potential remains underexplored. From basic research on health and diseases in the initial stage to the later focus on individual patients, these concepts have remained at the descriptive level. In future studies, it is necessary to leverage multidisciplinary theories and longitudinal tracking methods to deeply analyze the impact mechanisms of widowhood on the health, depression, and prevalence rates of older adults, and explore their application potential in practical interventions, thus advancing research from mere phenomenon description to mechanism - based explanation.

In summary, research on the mental health of widowed older adults should strengthen the guiding role of core themes, facilitate the integration of peripheral themes with core ones, increase investment in emerging areas, optimize research methods for declining fields, and deepen theoretical and practical explorations of fundamental core concepts. The above findings effectively respond to RQ2 and RQ3, elaborating on the current research status, hotspots, development trajectory and future research directions in this field.

### 4.4 Research hotspots

Through a comprehensive review of high-frequency keywords, highly cited literature, and cluster analysis, this study systematically depicts the research hotspots in the field of mental health among widowed older adults. The prominence of high-frequency keywords such as “health,” “older adults,” and “mental health” directly reflects the field’s intense focus on the overall health of older adults, particularly their mental well-being. The frequent appearance of keywords like “depression” and “prevalence” highlights the universality of depressive symptoms in widowed older adults and the broad research interest in this domain.

Highly cited literature has delved deeply into core issues of mental health among widowed older adults. *Widowhood and Depression: New Light on Gender Differences, Selection*, *and Psychological Adjustment (* 3) explores the mechanisms underlying depressive symptoms after spousal loss from the perspective of gender differences, providing a new research paradigm for understanding depression in this population. *Widowhood Status as a Risk Factor for Cognitive Decline among Older Adults (* 44) focuses on the association between widowhood and cognitive health, confirming that widowhood is a risk factor for cognitive decline and offering critical evidence for assessing the risk of cognitive impairment after spousal loss. As an authoritative diagnostic tool, the *Diagnostic and Statistical Manual of Mental Disorders (* 45) provides a standardized framework for accurately identifying depression, anxiety, and other mental disorders in widowed older adults. *Spousal Loss and Change in Cognitive Functioning: An Examination of Temporal Patterns and Gender Differences (* 48) reveals the trajectory of cognitive function changes over time after spousal loss through longitudinal tracking, providing empirical support for analyzing the dynamic impact mechanisms of widowhood on cognitive health. By exploring key areas such as depressive mechanisms, cognitive risks, diagnostic criteria, and dynamic changes, these studies not only lay the theoretical and methodological foundation for subsequent research but also highlight the centrality of research on the impact of widowhood on the psychological and cognitive health of older adults, as well as the sustained research enthusiasm in this field.

The results of cluster analysis further refine these research hotspots. Clusters such as “predictors,” “mental health,” “widowhood,” “survival analysis,” and “complicated grief” focus on specific aspects such as influencing factors of mental health, survival analysis, and complicated grief in widowed older adults. These clusters provide key directions for deeper exploration of the internal mechanisms and external manifestations of mental health in this population. Echoing the themes reflected by high-frequency keywords and highly cited literature, they collectively outline a comprehensive picture of the current research priorities in this field. These hotspots comprehensively address RQ2 by mapping current research priorities.

### 4.5 Research frontiers

The analysis of existing literature reveals a series of innovative and practically valuable research directions. The literature with the highest burst intensity focuses on psychological problems caused by widowhood, cognitive decline induced by widowhood, depression triggered by widowhood, and the impact of social and economic resources on the health of widowed older adults. This indicates that current research is transcending superficial understandings of mental health issues in widowed older adults, delving into the causal relationships between widowhood and specific psychological/cognitive impairments, as well as the regulatory mechanisms of socioeconomic factors on their health. High-frequency keywords with the strongest burst intensity, such as “loneliness,” “life course,” and “life satisfaction,” suggest that future research will place greater emphasis on developing intervention strategies targeting loneliness in widowed older adults. Studies will increasingly emphasize analyzing the long-term effects of widowhood on mental health from a dynamic life-course perspective while integrating life satisfaction into research frameworks to explore multidimensional approaches for enhancing mental health in this population.

Loneliness is one of the most prevalent psychological reactions among widowed older adults. Due to reduced social networks, broken interpersonal relationships, and lack of emotional support, it exerts profound impacts on mental health. Research shows that loneliness levels among widowed individuals are significantly higher than those among married individuals (16, 49). Additionally, interventions such as social support and cohabitation with family members have demonstrated significant effects in alleviating loneliness (49). Future research should deeply analyze the multidimensional mechanisms and longitudinal development trajectories of how loneliness influences the mental health of widowed older adults, while addressing its differential manifestations across cultural contexts. For example, in Chinese society, policies promoting traditional concepts of filial piety and family care models may effectively improve the well-being of widowed older adults. Furthermore, constructing multi-layered social support networks including community activities, volunteer programs, and virtual social platforms, along with providing personalized psychological counseling and emotional support, will help widowed older adults better cope with psychological challenges.

From a life-course perspective, widowhood represents a significant turning point in older adults’ lives. Intertwined with events such as retirement, deteriorating health, and family structure changes, it forms a complex stress chain. In the short term, it triggers intense psychological distress, while in the long term, it continuously influences mental health trajectories through chronic stress mechanisms. Future research could integrate the cumulative advantage and disadvantage model (50) to explore how socioeconomic resources exacerbate or mitigate widowhood-related psychological stress through cumulative effects. Mixed-method approaches including longitudinal studies and event sequence analysis are recommended to comprehensively examine the dynamic associations between widowhood and other major life events.

Life satisfaction, a key indicator reflecting older adults’ subjective evaluation of their life conditions, is influenced by multiple interacting factors such as social support, economic status, and health conditions (51). Widowed older adults commonly report lower life satisfaction, which is closely linked to their loneliness, health status, and coping strategies. Due to differences in cultural values and social environments, research findings on life satisfaction among widowed older adults vary significantly across countries and regions. Subsequent studies should comprehensively consider the complex factors affecting life satisfaction in this population, conduct longitudinal research to track its dynamic changes, and explore innovative intervention strategies such as mindfulness-based psychotherapy, expressive writing therapy, and community support programs.

These cutting-edge directions not only deepen academic understandings of mental health issues in widowed older adults but also provide new entry points for clinical interventions and social policy development, directly responding to Research Question 3 on future development trends and research agendas.

## 5 Conclusion

Through a systematic analysis of 891 relevant publications, this study constructs a global research landscape in the field of mental health among widowed older adults over the past 20 years, revealing the evolution of research hotspots and scientific development prospects. On one hand, the study finds that academic attention in this field has shown a continuous upward trend, particularly surging significantly after the COVID-19 pandemic, highlighting the urgency of mental health issues for special vulnerable groups. On the other hand, the exacerbation of population aging and the long-term nature of mental health problems have made the needs of widowed older adults a key focus of interdisciplinary research in global public health and social sciences. This study provides a panoramic analytical framework based on big data for the field, calls on academia to strengthen sustained attention to the mental health of widowed older adults, and its conclusions can also provide a scientific basis for policymakers to design targeted social support systems.

## 6 Limitation and future research

This study has several significant limitations. Regarding data sources and research scope, due to the incapability of bibliometric software, such as Citespace and VOSviewer, to effectively integrate multi-source data, especially data containing multilingual publications, the research solely relied on the Web of Science database. This choice introduced substantial selection bias, excluding a large amount of non-English literature and research findings from other regions. The Web of Science predominantly indexes research from Western academic communities, overlooking valuable studies conducted in other regions and languages. Consequently, the study failed to comprehensively consider the differences in research focus, methodology, and findings across various databases, narrowing the analytical scope and impeding a complete understanding of the global research landscape in the mental health of widowed older adults. Additionally, by concentrating solely on journal articles and reviews while excluding other scholarly outputs, such as book chapters and conference proceedings, the comprehensiveness of the research was further limited. Book chapters often contain in-depth theoretical discussions and comprehensive reviews that could enrich the understanding of the research topic, and conference proceedings may capture emerging research trends and preliminary findings, omission of which caused the study to miss out on novel perspectives and ongoing research directions.

In terms of research methods, the lack of author-level productivity analysis rendered the application of Lotka’s Law impossible. Moreover, the longitudinal modeling of cross-disciplinary publishing dynamics required by Price’s Law was beyond the scope of this study, making it difficult to gain in-depth insights into author contribution patterns and the growth laws of academic knowledge. Additionally, although analyzing the funding model and its correlations with macro indicators, such as GDP and global health indices, would be of great value, the Web of Science Core Collection has only systematically indexed funding metadata since 2008, resulting in the absence of early data during the study period. Furthermore, existing bibliometric tools are unable to effectively extract and analyze funding information, hindering the progress of this research direction.

To address these limitations, future research should strive to expand data sources by integrating multilingual databases and incorporating diverse scholarly outputs. Researchers should also utilize advanced tools to conduct author productivity analysis and publishing dynamics modeling, and strengthen cooperation with funding agencies to improve the mining and analysis of funding data. These efforts will contribute to a more comprehensive and in-depth understanding of the research landscape in the mental health of widowed older adults.

## Data Availability

The original contributions presented in the study are included in the article/Supplementary Material. Further inquiries can be directed to the corresponding authors.
